# Primary Charge Separation in the Photosystem II Reaction Center Revealed by a Global Analysis of the Two-dimensional Electronic Spectra

**DOI:** 10.1038/s41598-017-12564-4

**Published:** 2017-09-27

**Authors:** Hong-Guang Duan, Valentyn I. Prokhorenko, Emilie Wientjes, Roberta Croce, Michael Thorwart, R. J. Dwayne Miller

**Affiliations:** 1 Max Planck Institute for the Structure and Dynamics of Matter, Luruper Chaussee 149, 22761 Hamburg, Germany; 2 I. Institut für Theoretische Physik, Universität Hamburg, Jungiusstraße 9, 20355 Hamburg, Germany; 3 The Hamburg Center for Ultrafast Imaging, Luruper Chaussee 149, 22761 Hamburg, Germany; 4 Department of Physics and Astronomy and Institute for Lasers, Life and Biophotonics, Faculty of Sciences, VU University Amsterdam, De Boelelaan, 1081, 1081, HV, Amsterdam, The Netherlands; 5 The Departments of Chemistry and Physics, University of Toronto, 80 St. George Street, Toronto, M5S 3H6 Canada; 6 Present Address: Laboratory of Biophysics, Wageningen University, P.O. Box 8128, 6700 ET, Wageningen, The Netherlands

## Abstract

The transfer of electronic charge in the reaction center of Photosystem II is one of the key building blocks of the conversion of sunlight energy into chemical energy within the cascade of the photosynthetic reactions. Since the charge transfer dynamics is mixed with the energy transfer dynamics, an effective tool for the direct resolution of charge separation in the reaction center is still missing. Here, we use experimental two-dimensional optical photon echo spectroscopy in combination with the theoretical calculation to resolve its signature. A global fitting analysis allows us to clearly and directly identify a decay pathway associated to the primary charge separation. In particular, it can be distinguished from regular energy transfer and occurs on a time scale of 1.5 ps under ambient conditions. This technique provides a general tool to identify charge separation signatures from the energy transport in two-dimensional optical spectroscopy.

## Introduction

Photosystem II (PSII) is a unique biological system that is capable of separating electronic charge for the oxidation of water to oxygen using sunlight energy. The molecular structure of the PSII reaction center complex, which is composed by the D1/D2/cytb559 proteins, has been carefully resolved by X-ray crystallography^1^. It encompasses eight cofactors which are embedded in the protein: two primary chlorophylls *P*_*D*1_ and *P*_*D*2_, two accessory chlorophylls *Chl*_*D*1_ and *Chl*_*D*2_, two pheophytins *Pheo*_*D*1_ and *Pheo*_*D*2_, and two peripheral chlorophylls *Phlz*_*D*1_ and *Phlz*_*D*2_. All the cofactors are located symmetrically along the central axis of the D1–D2 reaction center complex and are classified into the D1 and the D2 branch. Only the active D1 branch is responsible for the charge separation (CS)^2^. Due to its importance, the PSII reaction center^3^ has been extensively studied over many years by almost all the available steady-state spectroscopic techniques, such as absorption, linear and circular dichroism (CD) and fluorescence spectroscopy^4,5^. In order to understand the mechanism of charge separation and transfer in the reaction center, photon-echo and transient absorption measurements have been combined for a more complete picture of the operating dynamics^6^. Based on the theoretical description of the kinetics resolved by the photon-echo spectra of the D1/D2-cytb559, it has been suggested that the fast primary CS process (${(Ch{l}_{D1}Phe{o}_{D1})}^{\ast }\to Ch{l}_{D1}^{+}Phe{o}_{D1}^{-}$) occurs on the timescale of 1.5 ps, while the much slower secondary CS process ($Ch{l}_{D1}^{+}Phe{o}_{D1}^{-}\to {P}_{D1}^{+}Phe{o}_{D1}^{-}$) was estimated to occur within 25 ps. Moreover, the time-resolved electron transport in the intact PSII core (composed of PSII RC complex, the inner antennas CP43 and CP47 and the oxygen evolving complex) has been studied by transient absorption spectroscopy and compared to the kinetics of the isolated D1/D2-cytb559 PSII reaction center^7^. Different timescales of the primary (5.5 ps) and secondary CS (35 ps) were revealed in the PSII core. In addition, a slow component with a time constant of 200 ps was resolved and was attributed to the electron transfer from cofactors to the *Q*_*A*_ acceptor. In these studies, the pigment *Chl*_*D*1_ is denoted as the electron donor of the initial CS process. In a later work, the D1/D2-cytb559 complex was investigated again by transient absorption spectroscopy with a large detection window of 3 ns^8^. The measured data have been analyzed by global and target analysis and it has been suggested that at least two primary CS pathways exist with time scales of 400 fs and 1.8 ps. The slow component with 1.8 ps is close to the previously mentioned value^6^ of 1.5 ps. Due to the better temporal resolution, the fast 400 fs component was resolved for the first time and attributed to CS. The associated theory suggested that it may be related to the CS in the radical pair. In addition to the short components, two other components were resolved which have much longer lifetimes (65 and 585 ps) and which were attributed to the secondary CS. The theoretical study proposed that strong static disorder affects the charge separated states and induces multiple CS processes^8^.

Among the available experimental techniques, two-dimensional (2D) electronic spectroscopy is arguably the most suitable tool to study the kinetics of the energy transfer (ET) in the photosynthetic complex^9^. The explicit use of a two pulse excitation sequence enables the identification of the inhomogeneous site distribution. In addition, it allows for a direct tracking of the energy and charge transfer pathway and the coherent transfer dynamics as a function of absorption and emission spectra^10^. These features are particularly useful in examining the photosynthetic system because the manifold of the electronic states is rather dense and additional considerable broadening due to static disorder yields highly congested spectra. The first experimental 2D spectroscopic study of the PSII reaction center was carried out at a temperature of 77 K^11^. Several relevant dynamical processes were identified on the basis of fitting each individual trace in the 2D map along the waiting time. Due to the mixed signal of the energy transfer and CS, there still lacks an effective tool to distinguish these two processes in the reaction center. The associated modeling of the CS state included an additional exciton state together with a larger disorder^12^. In addition, a tight-binding model has been used to describe the electron and hole interactions in the CS states^13,14^. Moreover, the long-lived vibronic quantum coherence has recently been reported to occur in the 2D spectroscopy of the PSII reaction center^15,16^. It was suggested that the CS processes are strongly related to these long-lived quantum oscillations, especially when the frequency of these coherent oscillations is resonant with the excitonic energy gap^17^. However, despite this significant progress of understanding CS in reaction center, direct evidence for the kinetics of CS in the PSII reaction center under ambient condition is still missing.

Here, we close this gap and explicitly reveal the CS dynamics of the PSII reaction center by 2D electronic spectroscopy at ambient temperature. We provide clear and direct evidence about the kinetics of the primary CS by comparing the experimental data with theoretical modeling. Two different CS components with the time constants <200 fs and ~1.5 ps are well resolved by the decay-associated spectra in a global fitting analysis. The CS dynamics can be clearly identified as a pair of a positive and a negative peak in the decay-associated spectra. We show that this feature can be straightforwardly understood already on the basis of a dimer model.

## Results

### Two-dimensional electronic spectra at ambient temperature

**Figure 1:**
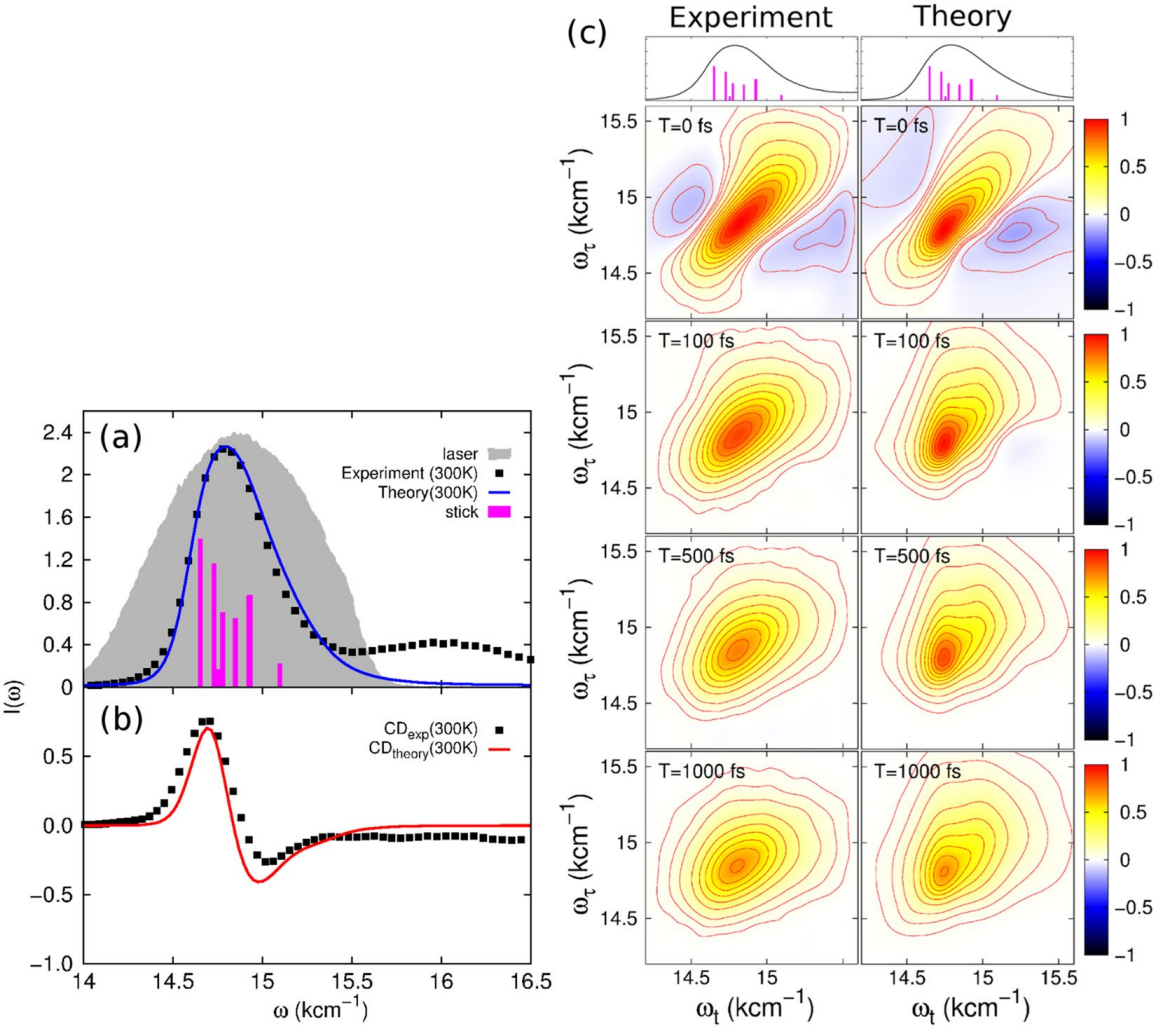
(**a**) Linear absorption spectrum of the PSII RC as experimentally measured (black symbols) and theoretically calculated (blue line) together with the stick spectrum (purple columns) and the spectrum of the laser pulse (grey shaded area) used in the experiment at room temperature. (**b**) The circular dichroism spectrum as measured (black symbols) and calculated (red line) with the same parameters as used in (**a**). (**c**) Real part of the experimental (left) and theoretical (right) 2D photon echo spectra of the PSII reaction center at different waiting times. The theoretical result is calculated with the model parameters obtained by fitting the linear absorption and CD spectra.

The details of the experimental setup, the sample preparation, and the measuring conditions are described in the Methods Section. The representative experimental 2D spectra (real part) are shown in the left column of Fig. 1(c) for the waiting times *T* = 0 fs, 100 fs, 500 fs and 1 ps. To obtain a dynamical modeling of the CS, we have used a tight-binding model (see below) and show the theoretical results for the 2D spectra in the right column of Fig. 1(c) for the same waiting times. Both results agree well, which demonstrates the validity of our theoretical model (see below). At *T* = 0 fs, the 2D spectra are significantly stretched along the diagonal direction which indicates strong inhomogeneous broadening in the PSII reaction center. The anti-diagonal cross section in 2D spectrum is related to the homogeneous broadening, which is determined by the fast electronic dephasing. The width of the anti-diagonal section is determined to be 56 fs, which is rather close to that of 62 fs for the LHCII^18^. Furthermore, two negative-amplitude cross peaks at initial time reveal absorption processes to doubly excited states. They disappear very quickly within a time window of <50fs. At *T* = 1 ps, both the experimental and theoretical 2D spectra still show evidence of the considerable inhomogeneous broadening. Evidence for the CS can be obtained from decay-associated spectra (see below). However, for their understanding, we need to set-up a tractable modeling and fitting procedure to the experimental data.

### Modeling charge separation in the reaction center

We use a tight-binding Hamiltonian^13^ to describe the electrons and holes which reside on different molecular parts and include their interaction as well. The pigment is represented by two orbitals, the highest occupied molecular orbital (HOMO) and the lowest unoccupied molecular orbital (LUMO). For the model, we define the operators ${\hat{e}}_{m}^{\dagger }$ (${\hat{e}}_{m}$) and ${\hat{h}}_{m}^{\dagger }$ (${\hat{h}}_{m}$), which create (annihilate) an electron in the LUMO or a hole in the HOMO of the pigment *m*, respectively. The operators satisfy Fermi commutation relations $\{{\hat{e}}_{m}^{\dagger },{\hat{e}}_{n}\}={\hat{e}}_{m}{\hat{e}}_{n}^{\dagger }+{\hat{e}}_{n}^{\dagger }{\hat{e}}_{m}={\delta }_{mn}$, $\{{\hat{h}}_{m}^{\dagger },{\hat{h}}_{n}\}={\hat{h}}_{m}{\hat{h}}_{n}^{\dagger }+{\hat{h}}_{n}^{\dagger }{\hat{h}}_{m}={\delta }_{mn}$, respectively. When the electron and hole reside on the same pigment *m*, we define a Frenkel-exciton (FE) state $|{m}^{\ast }\rangle ={\hat{e}}_{m}^{\dagger }{\hat{h}}_{m}^{\dagger }|g\rangle$, where $|g\rangle$ is the ground state of the pigment. When the electron and the hole reside at different pigments *m* and *n*, we have the charge separated state $|{n}^{+}{m}^{-}\rangle ={\hat{e}}_{m}^{\dagger }{\hat{h}}_{n}^{\dagger }|g\rangle$. With this, the transfer of the excitations and the charges as well as the Coulomb interaction between the charges and the (permanent) electric dipoles can be described in a transparent manner. The molecular Hamiltonian in the tight-binding form is given in the Methods Section.

The Hamiltonian matrix elements for the submanifold of the singly excited states is easily calculated. The excitation energy of the FE state m* follows as ${\varepsilon }_{{m}^{\ast }}={t}_{mn}^{e}+{t}_{mn}^{h}-{V}_{mm}^{eh}$. The excitation energy of the charge separated state *m* ^+^ *n* ^−^ is obtained as ${\varepsilon }_{{m}^{+}{n}^{-}}={t}_{nn}^{e}+{t}_{mm}^{h}-{V}_{nm}^{eh}$. The coupling between the FE states m* and n* amounts to ${J}_{{m}^{\ast },{n}^{\ast }}={W}_{mn}^{d}$ and the coupling between the FE state m* and the charge separated state *n* ^+^ *k* ^−^ is ${J}_{{m}^{\ast },{n}^{+}{k}^{-}}={t}_{mk}^{e}{\delta }_{mn}+{t}_{mn}^{h}{\delta }_{mk}$. Finally, the coupling between the charge separated states *m* ^+^ *n* ^−^ and *k* ^+^ *l* ^−^ reads ${J}_{{m}^{+}{n}^{-},{k}^{+}{l}^{-}}={t}_{nl}^{e}{\delta }_{mk}+{t}_{mk}^{h}{\delta }_{nl}$. In our theoretical modeling, we include the four primary charge separated states^19^ ${P}_{D2}^{+}{P}_{D1}^{-}$, $Ch{l}_{D1}^{+}Phe{o}_{D1}^{-}$, ${P}_{D1}^{+}Ch{l}_{D1}^{-}$ and ${P}_{D1}^{+}Phe{o}_{D1}^{-}$. The parameters are carefully chosen from the literature^12,14,20,21^ and fit to our experimental data (see Methods for details). The site energies and coupling terms of the double excitation manifold are described in the Supplementary Information (SI).

The modeling also encompasses coupling to a dissipative environment provided by the molecular vibrations (phonons) and the dielectric solvent. With the standard formalism of quantum dissipative systems, we consider a continuous distribution of fluctuating harmonic modes, which can be modeled as a bath of harmonic oscillators, ${H}_{B}={\sum }_{mj}({\omega }_{mj}{\hat{p}}_{mj}^{2}+{\hat{x}}_{mj}^{2})$. Here, the simplest linear system-bath interaction is taken into account by the coupling Hamiltonian ${H}_{SB}=-{\sum }_{mj}[{d}_{mj}^{e}{\hat{x}}_{mj}{\hat{e}}_{m}^{\dagger }{\hat{e}}_{m}+{d}_{mj}^{h}{\hat{x}}_{mj}{\hat{h}}_{m}^{\dagger }{\hat{h}}_{m}]$ where ${\hat{p}}_{j}$ and ${\hat{x}}_{j}$ are the dimensionless momentum and the corresponding dimensionless coordinate of the *j*-th bath mode, respectively. The bath spectral density has the form ${J}_{m}(\omega )=\frac{\pi }{2}{\sum }_{j}{d}_{mj}^{2}\delta (\omega -{\omega }_{mj})$. We assume a continuous distribution of the frequencies in the form of an Ohmic spectral density $J(\omega )=\gamma \omega {e}^{-\omega /{\omega }_{c}}$ (for FE: *γ* = 0.68, *ω*_*c*_ = 350 cm^−1^. For CS: *γ* = 1.36, *ω*_*c*_ = 350 cm^−1^). Here The validity of this choice is ensured by a careful fitting to our experimental data (see below). The parameters of the Ohmic spectral density were fitted by assuming three artificial spectral densities, thereby using Eq. (15) of ref.^22^. This approach significantly speeds up the numerical calculations.

Based on this model, the quantum dynamical calculations are carried out using the time non-local (TNL) quantum master equation^22,23^ (further details can be found in Supplementary Information). Inhomogeneous broadening of the spectral signals is generated by low frequency nuclear motion and static disorder, which is accounted for by an ensemble average over site energies with a Gaussian probability distribution (FWHM of 70 cm^−1^ for the FE states and 200 cm^−1^ for the charge separated states). In particular, to account for the spatially correlated disorder and fluctuations of the charge separated states, the Cholesky decomposition scheme is used to generate cross-correlated heat baths and static disorders^24^ (see the Supplementary Information for details). The strengths and the orientations of electric dipoles of the chromophores are taken from the direction of the NB–ND atom on the pigments (file 3ARC.pdb). The relative strengths of the transition dipole moments of the chlorophylls and the pheophytins molecules are set to |*μ*_*chl*_| = 1.0 and |*μ*_*Pheo*_| = 0.773^19^, respectively.

Equipped with this model, we have calculated the linear absorption and the circular dichroism spectra (for their detailed definition, see the Supplementary Information) and compare the results to the experimental data in Fig. 1(a) and (b). We find very good agreement between the experimental data and the parametrized model. The calculated stick spectrum of the PSII reaction center involves seven quantum states and is presented as well in Fig. 1a). Having fixed the model parameters, we then have calculated the 2D electronic spectrum using the equation of motion-phase matching-approach^25^. This yields the 2D photon echo spectra shown in the right column of Fig. 1(c). They agree very well with the experimental 2D spectra.

### Charge separation versus energy transfer: the decay-associated spectra

The signature of the CS can be clearly identified by analyzing the 2D decay-associated spectra (2DDAS), obtained by applying a multidimensional global fit approach to the series of consecutive 2D photon echo spectra acquired at different waiting times. The sequence of experimental 2D spectral data *S*(*ω*_*τ*_, *ω*_*t*_, *T*), recorded at different waiting times *T*, can be decomposed into several exponential components according to

1 $$\begin{array}{l}S({\omega }_{\tau },{\omega }_{t},T)=\sum _{i}{A}_{i}({\omega }_{\tau },{\omega }_{t})\exp (-T/{\tau }_{i}),\end{array}$$

where *A*_*i*_(*ω*_*τ*_, *ω*_*t*_) is the amplitude spectrum of single-exponential decay component associated with the decay timescale *τ*_*i*_. Thus, a positive peak at (*ω*_*τ*_, *ω*_*t*_) in the amplitude spectrum indicates an exponential decay of the magnitude in the 2D spectra (*ω*_*τ*_, *ω*_*t*_) along the waiting time with the time scale of *τ*_*i*_. In contrast, a negative peak denotes the exponential increase of the magnitude in 2D spectra with time scale of *τ*_*i*_. Based on these amplitude spectra, we retrieve the global kinetics of the 2D electronic spectra and how it evolves with the waiting time. The key properties of the DAS is to identify the ET and CS in the form of separated positive and negative peaks (see the explanation below). We use this tool to identify CS processes in the PSII reaction center.

**Figure 2:**
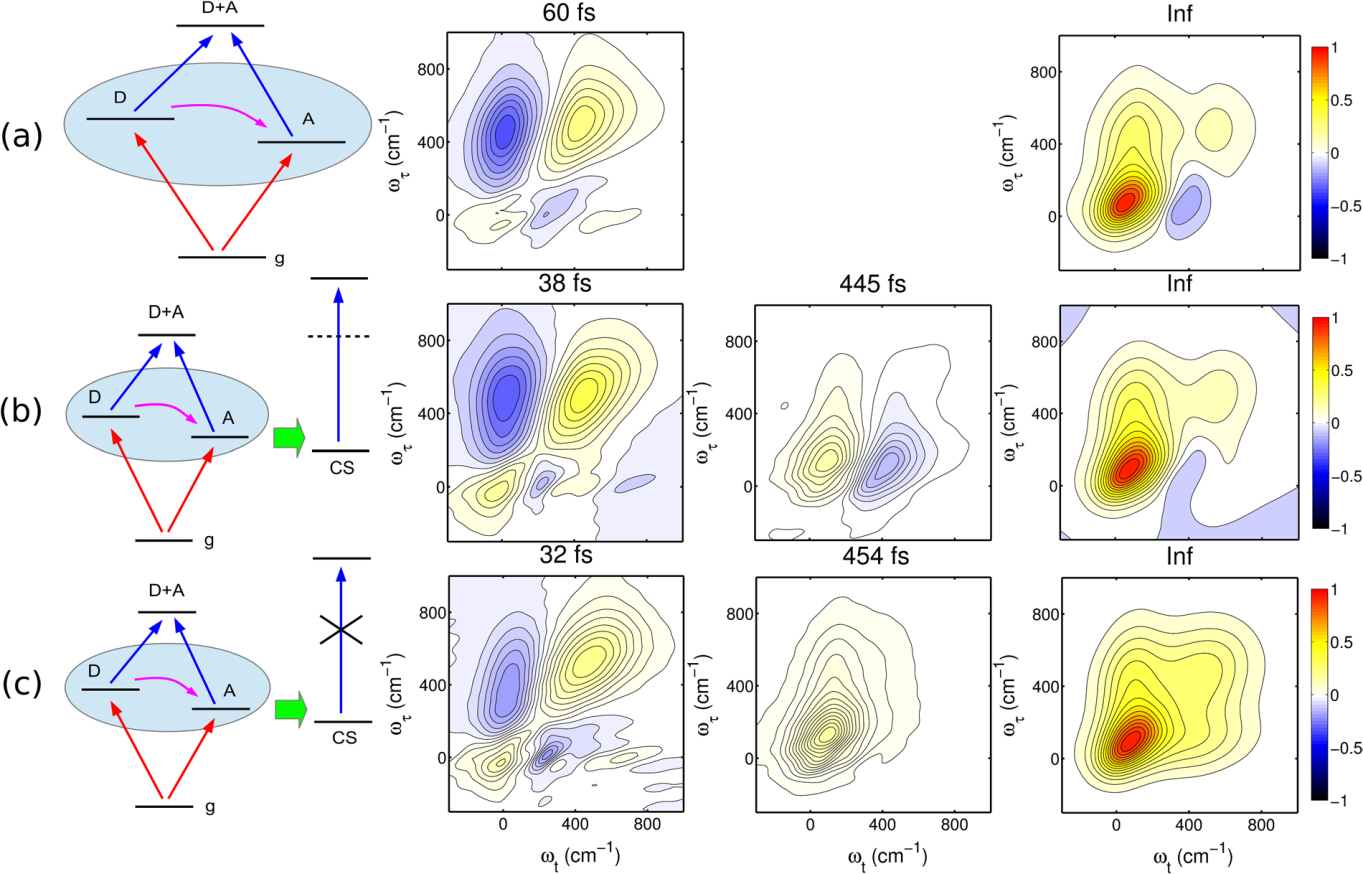
Decay-associated spectra of the three variants of the dimer model. (**a**) Pure excitonic dimer without the charge separated state. (**b**) Dimer with one charge separated state *D* ^+^ *A* ^−^. (**c**) Dimer model with one charge separated state, but the transition to the charge-transfer-related double excited states is artificially excluded.

In order to illustrate how ET can be discriminated from CS by the 2DDAS, let us first consider a simple dimer model of a donor (D)-acceptor (A) pair. For this reference case, the site energies are chosen as *ε*_1_ = 270 cm^−1^ and *ε*_2_ = 210 cm^−1^, respectively. The excitonic coupling between the monomers is set to *J* = 150 cm^−1^. No charge separated state is included. In the same way as described above for the PSII reaction center, the 2D photon echo spectra of the dimer are calculated for waiting times up to 5 ps. Then, the kinetics and the 2DDAS are extracted by applying a multidimensional global fit approach to the series of consecutive 2D spectra acquired at different waiting times. The result for the 2DDAS of the dimer without the charge separated state is shown in Fig. 2a). Clear evidence of the ET in the first 2DDAS associated with the decay time constant of 60 fs appears as one diagonal positive peak located at 400 cm^−1^. In addition, two negative cross peaks are found at *ω*_*t*_ = 0 cm^−1^, *ω*_*τ*_ = 400 cm^−1^ (upper left) and *ω*_*t*_ = 400 cm^−1^, *ω*_*τ*_ = 0 cm^−1^ (lower right). It indicates that in the 2D photon echo spectra, the magnitude of the diagonal peak at *ω*_*t*_ = *ω*_*τ*_ = 400 cm^−1^ follows an exponential decay with the corresponding time constant 60 fs. Likewise, the magnitudes of the upper left and lower right cross peaks follow an exponential increase with the same time constant. In addition, the upper left cross peak has a much larger magnitude and thus a much stronger increase than the lower right cross peak. This observation is a manifestation of the dominant “downhill” ET between the two excitonic states. The asymptotic steady state component with the longest time constant (infinity) is shown in Fig. 2(a) and marked by “Inf”.

To illustrate the signature of the CS, we add one charge separated state to the dimer in the form *D* ^+^ *A* ^−^ with the CS direction *D* → *A* (see the Supplementary Information for the Hamiltonian) and calculate the 2D photon echo spectra again for the first 5 ps of the waiting time. From these, we extract the 2DDAS for which its lifetime components are shown in Fig. 2b). In addition to the ET component with a time constant of 38 fs, we find one special CS component with a time constant of 445 fs. The CS component exhibits a positive peak and a negative peak located at the two opposite sides of the diagonal axis. The position of the negative peak at *ω*_*t*_ = 400 cm^−1^, *ω*_*τ*_ = 100 cm^−1^ is the same as the location of the double excited states in the 2D photon echo spectrum. This implies that the magnitude of the double excited states increases for increased waiting times. This can be uniquely traced back to the interaction of the permanent dipoles^26^ which is included by the last term (~*K*_*kl*,*mn*_) in the tight-binding Hamiltonian in Eq. (3). To further clarify this feature, we deliberately remove the transition to the CS-related double excited state in the Hamiltonian and calculate the 2D photon echo spectra of this “quenched” dimer. The resulting 2DDAS yields the decay-associated life time components shown in Fig. 2c). We clearly observe that one component with the comparable lifetime of 454 fs appears and shows the same characteristics of the CS signature as in Fig. 2b), but without the negative peak. This confirms that the pair of one positive and one negative cross-peak aside from the diagonal in the 2DDAS is associated to the existence of the CS mechanism. In passing, we note that in a similar way, multiple CS pathways can be identified. We have calculated the kinetics and the 2D photon spectra of a tetramer model in which two charge separated states were included in the model. Two CS components can also be clearly resolved and different lifetimes can be identified (for details, see the Supplementary Information).

### Charge separation in the reaction center

**Figure 3:**
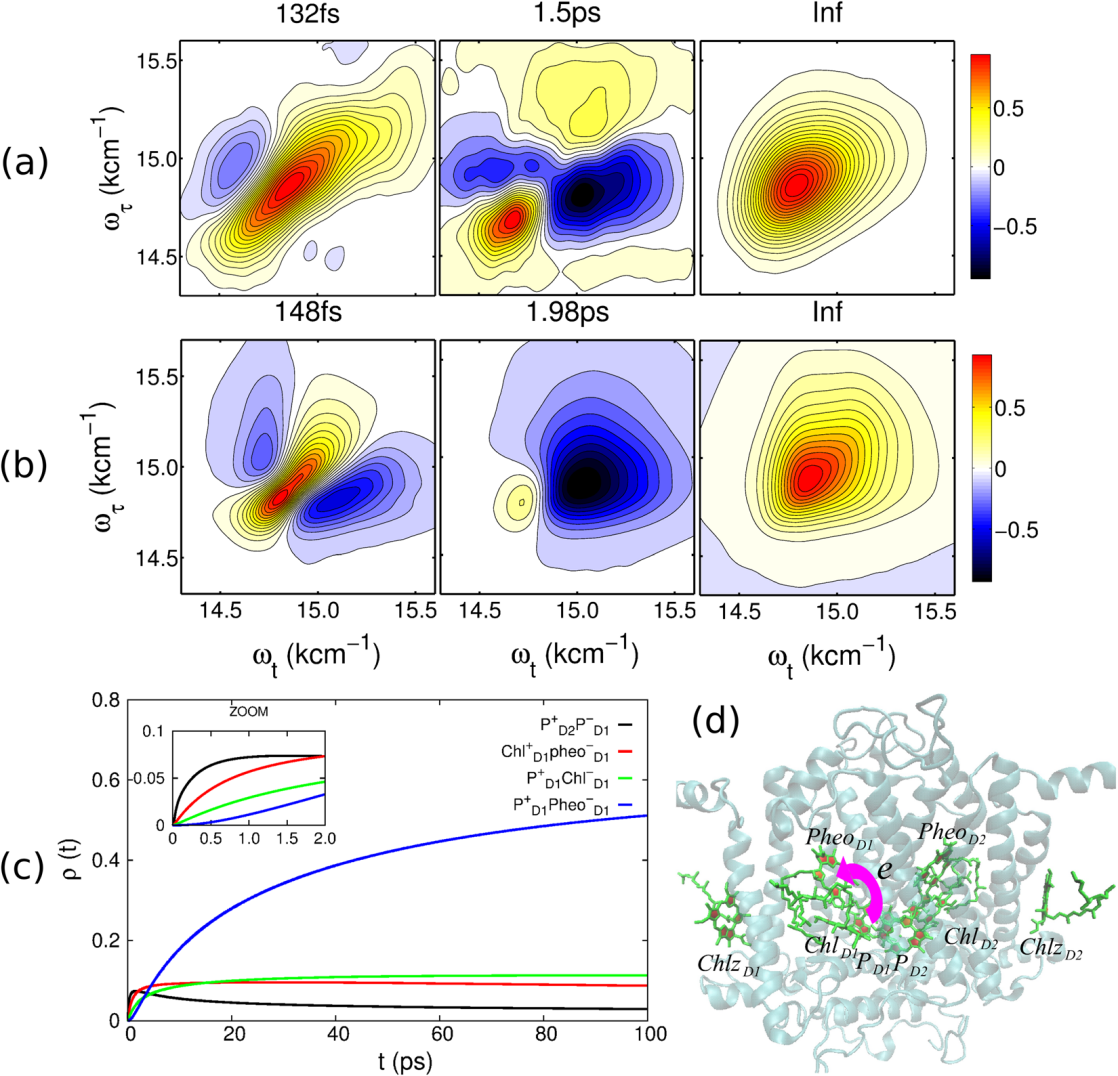
Decay-associated spectra of the PSII reaction center. (**a**) Experimental data. (**b**) Theoretical result. (**c**) The calculated population dynamics of the charge separated states. For the calculation, the initial population of each pigment is assigned according to the magnitude of its transition dipole moment. (**d**) Molecular structure of the PSII reaction center complex, which is composed by the D1/D2/cytb559 proteins.

Having revealed the basic mechanism and the signature of the CS in the 2DDAS, we next investigate the CS in the PSII RC complex. For this, we analyze the experimental 2D photon echo spectra depicted in Fig. 1c) by the multidimensional global fitting approach. The resulting lifetime components are shown in Fig. 3a). We find three different time constants, i.e., 132 fs, 1.5 ps and 13.9 ps (see the Supplementary Information for the last case). The theoretically calculated 2D photon echo spectra can be analyzed in the same way. We find the theoretically determined 2DDAS as shown in Fig. 3b) with the lifetime components 148 fs and 1.98 ps. The third component of approximately 14 ps does not appear in the theoretical spectra due to the limited delay time window used for calculations (6 ps).

For the shortest experimental lifetime component of 132 fs, a broadband positive peak is located at *ω*_*t*_ = *ω*_*τ*_ = 14800 cm^−1^ along the diagonal direction, which we attribute to fast electronic dephasing. In addition, a less pronounced negative cross peak at *ω*_*t*_ = 14700 cm^−1^, *ω*_*τ*_ = 15000 cm^−1^ indicates the growth of the amplitude of this feature. As demonstrated for the dimer test model, this is a typical signature of ET. The corresponding shortest lifetime component of 148 fs is resolved from the theoretical part as well. It shows a positive peak of fast electronic dephasing located on the diagonal at *ω*_*t*_ = *ω*_*τ*_ = 14800 cm^−1^. In addition, two negative cross peaks appear. One fast ET component is revealed at *ω*_*t*_ = 14800 cm^−1^, *ω*_*τ*_ = 15000 cm^−1^. In addition, a CS component is uncovered by the lower right negative cross peak at *ω*_*t*_ = 15200 cm^−1^, *ω*_*τ*_ = 14800 cm^−1^. This is rationalized by resolving the 2DDAS components calculated with the dimer model plus one additional charge separated state (see above). It is different in the experimental 2DDAS, where the fastest lifetime resolved from the calculated spectra (148 fs) is associated with the mixing of electronic dephasing, fast ET and CS. This CS component could be associated with the rapid formation of the charge separated state (*P*_*D*1_ *P*_*D*2_)* → ${P}_{D1}^{+}{P}_{D2}^{-}$ as revealed by the calculated spectra. However, the suppression of this CS component in the current room-temperature experimental data is likely to be due to high-frequency (fast) noise in the experiment.

In addition to the above, a CS component with a time constant of 1.5 ps is well resolved in the experimental data. The strong negative peak is located at (*ω*_*τ*_, *ω*_*t*_) = (14750 *cm* ^−1^, 15000 cm^−1^), which agrees with the resolved negative peak in the right-down section of the DAS in the dimer model (Fig. 2). The theoretical result shows good agreement of the negative peak with the experimental result, with the resulting life time of 1.98 ps. This life time component fits well with the rate of the process of the primary CS between the electronic donor and acceptor and is associated to the process ${(Ch{l}_{D1}Phe{o}_{D1})}^{\ast }$ → $Ch{l}_{D1}^{+}Phe{o}_{D1}^{-}$ revealed by dynamical calculations of the charge separated states (see Fig. 3(c) and (d)). In addition, the spectral difference is still presented in this component after comparing the experimental result with the theoretical prediction. A further improvement requires a refined modeling which goes beyond the scope of the present work. A possible route would be to use a very advanced atomic *ab-initio* modeling of the 2D electronic spectrum^27,28^.

Moreover, a well-resolved lifetime component with 13.9 ps shows up (see the Supplementary Information) with only one diagonal positive peak located at *ω*_*t*_ = *ω*_*τ*_ = 14800 cm^−1^. Within our present calculations, it cannot be revealed by the theoretical part since only rather short waiting times up to 6 ps could be calculated, which is not enough to resolve the 14 ps lifetime component (see details of the highly parallelized message-passing-interface calculations in the Supplementary Information). In any case, this component does not show any typical signatures of ET or CS. We interpret it as slow energy transport from two peripheral chlorophylls (*Chlz*_*D*1_ and *Chlz*_*D*2_) to the central cofactors due to rather weak electronic couplings arising from the relatively large distance involved in the transport.

In addition to the primary CS, secondary CS processes i.e.,

2 $$\begin{array}{c}{(Ch{l}_{D1}Phe{o}_{D1})}^{\ast }\to Ch{l}_{D1}^{+}Phe{o}_{D1}^{-}\,\to\,{P}_{D1}^{+}Phe{o}_{D1}^{-},\\ {({P}_{D1}{P}_{D2})}^{\ast }\to {P}_{D2}^{+}{P}_{D1}^{-}\,\to\,{P}_{D1}^{+}Ch{l}_{D1}^{-}\,\to\,{P}_{D1}^{+}Phe{o}_{D1}^{-}.\end{array}$$

Here, we did not resolve any component with a long lifetime which could be attributed to the secondary CS process denoted by the broken arrows in the sequence (2). The CS ${P}_{D2}^{+}{P}_{D1}^{-}$ → ${P}_{D1}^{+}Ch{l}_{D1}^{-}$ is not well resolved by the multidimensional global fitting since the weight factor of this CS pathway is small due to the relatively high site energy of ${P}_{D2}^{+}{P}_{D1}^{-}$ in the current model. However, this component can be well resolved in the dynamical calculations, see Fig. 3(c). One possible explanation for the appearance of the other CS components is that the secondary CS processes lasts longer than the experimentally accessible waiting-time window exceeding 80 ps realized in the experiment. Finally, in the theoretical modelings we do not include any vibrational mode, but only consider electronic features to simplify the model of theoretical calculations for the CS.

In conclusion, the CS dynamics of PSII reaction center leaves clear traces in the 2D photon echo spectroscopy. The CS can be uniquely and directly revealed by the global fitting analysis of the 2D spectra, which results in clearly resolved lifetime components in the 2D maps. In the study of the PSII reaction center, both the theoretical and the experimental data show very good agreement. This allows us to identify primary CS which occurs within 1.5 ps at ambient conditions.

## Methods

### Experimental setup

Two-dimensional electronic spectroscopy in the regime of visible light is well established^10,18^. As in previously reported experiments^18^, in order to suppress the excitation of high-lying vibrational states of chlorophylls and pheophytines, the spectrum of excitation laser pulses is explicitly restricted to the spectral region 630–720 nm.

### Sample preparation

Thylakoid membranes were isolated from A. thaliana plants as described in ref.^29^ till the centrifugation step at 6000 g. Thylakoid membranes were solubilized with 0.6% dodecyl-D-maltoside (DDM) at a final chlorophyll concentration of 0.5 mg/ml. The sucrose density ultracentrifugation was used to obtain PSII core particles as described in ref.^30^. The purification of PSII RCs from PSII core particles proceeds as follows: the PSII core particles were diluted in BTS200 buffer (20 mM Bis Tris pH 6.5, 20 mM MgCl2, 5 mM CaCl2, 10 mM MgSO4, 0.03% DDM, 0.2 M sucrose) to a chlorophyll concentration of 0.15 mg/ml and solubilized with an equal volume of 10% Triton X-100 in BTS200 buffer for 20 min; then the material was loaded on a HiTrap Q Sepharose HP 1 ml column (GE Healthcare) and washed with a BTS buffer until the eluate became colorless. Finally, the PSII RC particles were eluted from the column with 75 mM MgSO4 in a BTS200 buffer. The sample absorption spectrum in the Qy band is shown in Fig. 1a) together with the laser spectrum. The circular dichroism spectrum is depicted in Fig. 1b).

### Measuring conditions

Direct before measurements, the RC-sample was filtered using a 0.2-*μ*m micropore filter and placed into 0.8-mm thick closed cell with the inner volume of 2 mL, mounted onto a precise home-build X-Y motorized translator allowing a continuous changing of the excitation spot position. The spot size of all beams at the sample position was ~60 *μ*m. For minimizing unwanted contributions of non-resonant response to photon echo signal, the input cell window was made from a 150 *μ*m fused silica cover slip. To ensure annihilation-free excitation conditions, the energy of the laser pulses was kept below 5 nJ (except of the local oscillator beam which was attenuated with a neutral-density filter by a factor of 100).

The heterodyne-detected photon echo spectra were collected at each fixed waiting time *T* by scanning the *τ* delay in a range of [−100, +150] fs, with a delay time step of 1 fs. At each delay point 320–440 spectra were averaged (depending on *T*) to achieve a high SNR. The waiting time *T* was linearly scaled within the first 2 ps delay and then logarithmically-spaced up to 80 ps.

### Treatment of collected data

The measured data were processed according to the procedure described in detail in ref.^18^. The two-dimensional global analysis^31^ of the series of 2D spectra was performed using 4 life times and starting from a *T* = 25 fs delay.

### Theoretical modeling of the charge transfer

We have used a tight-binding model for the charge transfer in the reaction center. In terms of the creation (annihilation) operators ${\hat{e}}_{m}^{\dagger }$ (${\hat{e}}_{m}$) and ${\hat{h}}_{m}^{\dagger }$ (${\hat{h}}_{m}$) for an electron in the LUMO or a hole in the HOMO of the pigment *m*, the Hamiltonian reads^13,14^

3 $$\begin{array}{rcl}{H}_{S} & = & \sum _{m,n}{t}_{mn}^{e}{\hat{e}}_{m}^{\dagger }{\hat{e}}_{n}+\sum _{m,n}{t}_{mn}^{h}{\hat{h}}_{m}^{\dagger }{\hat{h}}_{n}+\sum _{m,n}^{m\ne n}{W}_{mn}^{d}{\hat{e}}_{m}^{\dagger }{\hat{h}}_{m}^{\dagger }{\hat{h}}_{n}{\hat{e}}_{n}\\  & - & \sum _{m,n}{V}_{mn}^{eh}{\hat{e}}_{m}^{\dagger }{\hat{h}}_{n}^{\dagger }{\hat{h}}_{n}{\hat{e}}_{m}+\frac{1}{2}\sum _{m,n}^{m\ne n}{V}_{mn}^{e}{\hat{e}}_{m}^{\dagger }{\hat{e}}_{n}^{\dagger }{\hat{e}}_{n}{\hat{e}}_{m}\\  & + & \frac{1}{2}\sum _{m,n}^{m\ne n}{V}_{mn}^{h}{\hat{h}}_{m}^{\dagger }{\hat{h}}_{n}^{\dagger }{\hat{h}}_{n}{\hat{h}}_{m}\\  & + & \frac{1}{4}\sum _{k,m}^{k\ne m}\sum _{l,n}^{l\ne n}{K}_{kl,mn}{\hat{e}}_{k}^{\dagger }{\hat{h}}_{l}^{\dagger }{\hat{e}}_{m}^{\dagger }{\hat{h}}_{n}^{\dagger }{\hat{h}}_{n}{\hat{e}}_{m}{\hat{h}}_{l}{\hat{e}}_{k}.\end{array}$$

Here, ${t}_{mn}^{e}$ and ${t}_{mn}^{h}$ are the hopping matrix elements between the LUMO and HOMO of different pigments, respectively. ${W}_{mn}^{d}$ describes the strength of the Coulomb interaction between the charges on the pigments *m* and *n* and ${V}_{mn}^{eh}$ is the electron-hole interaction between the charges on the pigments *m* and *n*. Likewise, ${V}_{mn}^{e}$ and ${V}_{mn}^{h}$ are the electron-electron and hole-hole Coulomb repulsion, respectively. The last term *K*_*kl*,*mn*_ represents the interaction energy of the permanent dipole moments of the two doubly excited states.

For the specific choice of the parameters, let us consider the elements of the Hamiltonian of the single excitation submanifold. First, the Coulomb interaction terms *J*_m*,n*_ between two FE states were calculated by the TrESP method^32^, where the Dexter-type exchange component was properly included in the interaction term for *P*_*D*1_ and *P*_*D*2_. We use those parameters without any further modifications. Second, the initial site energies *ε*_m*_ of the FE states are available^21^ and are, in addition, further optimized by the simultaneous fit of the linear absorption and the circular dichroism spectra at two different temperatures (77 K and 300 K). Third, the initial values of the site energies of the charge separated states and the corresponding interactions of the FE–charge separated and CS–CS states are extracted from ref.^19^, in which the site energies of the CT states were optimized by the simultaneous fitting of the charge-sensitive time-resolved fluorescence and Stark spectra^21^. The interaction strengths for the FE and charge separated states and the CS-CS states are determined by fitting the kinetics of the transient absorption spectra obtained with different wavelengths of the excitation.

## Electronic supplementary material

Primary Charge Separation in the Photosystem II Reaction Center Revealed by a Global Analysis of the Two-dimensional Electronic Spectra
