# Supplementary material for: Primary Charge Separation in the Photosystem II Reaction Center Revealed by a Global Analysis of the Two-dimensional Electronic Spectra
Source: Sci Rep. 2017 Sep 27;7:12347. doi: 10.1038/s41598-017-12564-4 (PMC5617839; doi:10.1038/s41598-017-12564-4)
Supplement: Supplementary file 1 — Primary Charge Separation in the Photosystem II Reaction Center Revealed by a Global Analysis of the Two-dimensional Electronic Spectra [file 41598_2017_12564_MOESM1_ESM.pdf]

Primary Charge Separation in the Photosystem II Reaction Center Revealed by a Global Analysis of the Two-dimensional Electronic Spectra

– Supplementary Information –

Hong-Guang Duan[1,2,3,*], Valentyn I. Prokhorenko[1,*], Emilie Wientjes[4,‡],
Roberta Croce[4], Michael Thorwart[2,3] & R. J. Dwayne Miller[1,3,5]
[1] *Max Planck Institute for the Structure and Dynamics of Matter, Luruper Chaussee 149, 22761, Hamburg, Germany*
[2] *I. Institut für Theoretische Physik, Universität Hamburg, Jungiusstraße 9, 20355 Hamburg, Germany*
[3] *The Hamburg Center for Ultrafast Imaging, Luruper Chaussee 149, 22761 Hamburg, Germany*
[4] *Department of Physics and Astronomy and Institute for Lasers, Life and Biophotonics, Faculty of Sciences, VU University Amsterdam, De Boelelaan, 1081, 1081, HV, Amsterdam, The Netherlands*
[5] *The Departments of Chemistry and Physics, University of Toronto, 80 St. George Street, Toronto Canada M5S 3H6*
[*] *These authors contributed equally to this work.*
[‡] *Present address: Laboratory of Biophysics, Wageningen University, P.O. Box 8128, 6700 ET Wageningen, The Netherlands*

I. MODEL HAMILTONIAN

The molecular structure of the photosystem II (PSII) reaction center (RC) has been resolved by X-ray crystallography and is well known [1–3]. It has eight cofactors embeded in a protein matrix. For the modeling, we describe the optical transition in each cofactor in terms of individual quantum two-level systems. We use the matrix elements of the electronic coupling between the pigments and the initial values of the site energies of Ref. [4]. We add four different charge separated (CS) states, denoted by $P^+_{D1}P^-_{D2}$, $Chl^+_{D1}Pheo^-_{D1}$, $P^+_{D1}Chl^-_{D1}$ and $P^+_{D1}Pheo^-_{D1}$. The coupling between the CS states were resolved by transient absorption spectroscopy [5, 6]. We assume the electronic coupling to be constant and further refine the site energies of the Frenkel exciton (FE) and the CS states by fitting the linear absorption and circular dichroism spectrum at different temperatures to the experimentally determined results. The final Hamiltonian matrix in the optimized form and the single-excited state manifold reads

$$\begin{bmatrix} 290 & 150 & -42 & -55 & -6 & 17 & 1 & 1 & 45 & 0 & 10 & 0 \\ 150 & 180 & -56 & -35 & 20 & -2 & 1 & 1 & 45 & 0 & 0 & 0 \\ -42 & -56 & 0 & 7 & 46 & -4 & 3 & 0 & 0 & 22 & 10 & 0 \\ -55 & -35 & 7 & 150 & -5 & 37 & 0 & 2 & 0 & 0 & 0 & 0 \\ -6 & 20 & 46 & -5 & 120 & -3 & -4 & 0 & 0 & 22 & 0 & 0 \\ 17 & -2 & -4 & 37 & -3 & 20 & 0 & -4 & 0 & 0 & 0 & 0 \\ 1 & 1 & 3 & 0 & -4 & 0 & 240 & 0 & 0 & 0 & 0 & 0 \\ 1 & 1 & 0 & 2 & 0 & -4 & 0 & 240 & 0 & 0 & 0 & 0 \\ 45 & 45 & 0 & 0 & 0 & 0 & 0 & 0 & 160 & 0 & 0 & 0 \\ 0 & 0 & 22 & 0 & 22 & 0 & 0 & 0 & 0 & 0 & 0 & 0 \\ 10 & 0 & 10 & 0 & 0 & 0 & 0 & 0 & 0 & 0 & -60 & 35 \\ 0 & 0 & 0 & 0 & 0 & 0 & 0 & 0 & 0 & 0 & 35 & -500 \end{bmatrix}. \tag{1}$$

All entries are given in units of cm^{-1} and have been shifted by 14700 cm^{-1} by the rotating-wave approximation. The associated index of Hamiltonian matrix are labeled as: P_{D1}, P_{D2}, Chl_{D1}, Chl_{D2}, $Pheo_{D1}$, $Pheo_{D2}$, $Chlz_{D1}$, $Chlz_{D2}$, $P^+_{D1}P^-_{D2}$, $Chl^+_{D1}Pheo^-_{D1}$, $P^+_{D1}Chl^-_{D1}$ and $P^+_{D1}Pheo^-_{D1}$.

In the double-excited manifold, the Hamiltonian matrix elements are given by

$$
\begin{aligned}
&\epsilon_{m^*n^*} = \epsilon_{m^*} + \epsilon_{n^*} + K_{mm,nn},\\
&\epsilon_{m^*n^+k^-} = \epsilon_{m^*} + \epsilon_{n^+k^-} + K_{mm,kn},\\
&\epsilon_{m^+n^-k^+l^-} = \epsilon_{m^+n^-} + \epsilon_{k^+l^-} + V^{e}_{nl} + V^{h}_{mk} - V^{eh}_{nk} - V^{eh}_{ml} + K_{nm,lk},\\
&J_{m^*n^*,m'^*n'^*} = J_{m^*,m'^*}(1-\delta_{mm'})\delta_{nn'} + J_{m^*,n'^*}(1-\delta_{mm'})\delta_{nm'} + J_{n^*,m'^*}(1-\delta_{nm'})\delta_{mn'} + J_{n^*,n'^*}(1-\delta_{nn'})\delta_{mm'},\\
&J_{m^*n^+k^-,m'^*n'^*} = \delta_{mm'} J_{m'^*,n^+k^-},\\
&J_{m^+n^-k^+l^-,m'^*n'^*} = 0,\\
&J_{m^*n^+k^-,m'^*n'^+k'^-} = \delta_{mm'} J_{n^+k^-,n'^+k'^-} + \delta_{nn'}\delta_{kk'}(1-\delta_{mm'})J_{m^*,m'^*} + \delta_{mn'}\delta_{nm'}\delta_{kk'}J_{m^*,m^+m'^-} + \delta_{mk'}\delta_{nn'}\delta_{km'}J_{m^*,m'^+m^-},\\
&J_{m^+n^-k^+l^-,m'^*n'^+k'^-} = \delta_{nk'}\delta_{kn'}J_{m'^*,m^+l^-} + \delta_{kn'}\delta_{lk'}J_{m'^*,m^+n^-} + \delta_{mn'}\delta_{lk'}J_{m'^*,k^+n^-} + \delta_{mn'}\delta_{nk'}J_{m',k^+l^-},\\
&J_{m^+n^-k^-l^-,m'^+n'^-k'^+l'^-} = \delta_{kk'}\delta_{ll'}J_{m^+n^-,m'^+n'^-} + \delta_{mm'}\delta_{nn'}J_{k^+l^-,k'^+l'^-} + \delta_{kk'}\delta_{ln'}J_{m^+n^-,m'^+l'^-} + \delta_{mm'}\delta_{nl'}J_{k^+l^-,k'^+n'^-}\\
&+ \delta_{mk'}\delta_{ll'}J_{k^+n^-,m'^+n'^-} + \delta_{km'}\delta_{ln'}J_{m^+n^-,k'^+l'^-} + \delta_{mk'}\delta_{nl'}J_{k^+l^-,m'^+n'^-} + \delta_{nn'}\delta_{km'}J_{m^+l^-,k'^+l'^-}\,.
\end{aligned}
\tag{2}
$$

Here, $\epsilon_{m^*n^*}$ is the site energy of the doubly excited state formed by the FE-FE states. $\epsilon_{m^*n^+k^-}$ and $\epsilon_{m^+n^-k^+l^-}$ are the site energies associated to the FE-charge separated states and the CS-CS states, respectively. $J_{m^*n^*,m'^*n'^*}$ is the electronic coupling of the FE-FE states between the states m^*n^* and $m'^*n'^*$. Moreover, $J_{m^+n^-k^+l^-,m'^*n'^*}$ is the coupling between the CS-CS state $m^+n^-k^+l^-$ and the FE-FE state $m'^*n'^*$. In addition, $J_{m^*n^+k^-,m'^*n'^+k'^-}$ is the coupling between the FE-CS states $m^*n^+k^-$ and $m'^*n'^+k'^-$, and $J_{m^+n^-k^+l^-,m'^*n'^+k'^-}$ is the coupling between the CS-CS state $m^+n^-k^+l^-$ and the FE-CS $m'^*n'^+k'^-$. Finally, $J_{m^+n^-k^+l^-,m'^+n'^-k'^+l'^-}$ is the coupling between the CS-CS states $m^+n^-k^+l^-$ and $m'^+n'^-k'^+l'^-$. In this calculation, we assume the $K_{mm,nn} = 0$, $K_{mm,kn} = 500$ cm^{-1} and $K_{nm,jk} = 2000$ cm^{-1}.

II. THEORETICAL METHODS FOR SPECTROSCOPIC CALCULATIONS

A. Time non-local quantum master equation

For the numerical simulations of the linear and 2D spectra, we have applied the time non-local (TNL) quantum master equation [7, 8]. The time evolution of the total density matrix $\rho(t)$ at time t which includes the system and the bath is governed by the Liouville-von Neumann equation with the Liouville superoperator $\mathcal{L}$, according to ($\hbar = 1$)

$$\dot{\rho} = -i[H_{\rm tot}, \rho] = \mathcal{L}\rho\,. \tag{3}$$

The total Hamiltonian $H_{\rm tot} = H_{\rm s} + H_{\rm b} + H_{\rm sb} + H_{\rm ren}$ includes the system, the bath, and the interaction and renormalization terms. For the system with a single degree of freedom x and the bath consisting of an ensemble of harmonic oscillators $H_{\rm b} = \sum_{j=1}^{N}\left[p_j^2/(2m_j) + m_j\omega_j^2 x_j^2/2\right]$, the coupling between them is assumed to be of the form $H_{\rm sb} = f(x)\sum_{j=1}^{N} c_j x_j$ with some real function $f(\cdot)$.

The projection scheme of Nakajima and Zwanzig [9] separates the dynamics of the system and the bath. The bath is assumed in a thermal state represented by the canonical density operator $\rho_{\rm b}^{\rm eq} = \exp(-\beta H_{\rm b})$ with a given temperature $T = (k_B\beta)^{-1}$. Applying the projector $P = \rho_{\rm b}^{\rm eq}{\rm tr}_{\rm b}$ with ${\rm tr}_{\rm b}\rho_{\rm b}^{\rm eq} = 1$ and its orthogonal complement $Q = 1 - P$ yields a formally exact quantum master equation for the time evolution of the reduced system density operator $\rho_{\rm s}(t) = {\rm tr}_{\rm b}\rho(t)$ [7] in the form of

$$
\begin{aligned}
\dot{\rho}_{\rm s}(t) &= \mathcal{L}_{\rm s}^{\rm eff}\rho_{\rm s}(t) + \int_0^t K(t,t')\rho_{\rm s}(t') + \Gamma(t),\\
\mathcal{L}_{\rm s}^{\rm eff} &= \mathcal{L}_{\rm s} + {\rm tr}_{\rm b}\mathcal{L}_{\rm sb}\rho_{\rm b}^{\rm eq} + \mathcal{L}_{\rm ren},\\
K(t,t') &= {\rm tr}_{\rm b}\mathcal{L}_{\rm sb}\left(\mathcal{T}e^{\int_{t'}^{t} Q\mathcal{L}dt''}\right)Q(\mathcal{L}_{\rm b} + \mathcal{L}_{\rm sb})\rho_{\rm b}^{\rm eq},\\
\Gamma(t) &= {\rm tr}_{\rm b}\mathcal{L}_{\rm sb}\left(\mathcal{T}e^{\int_{0}^{t} Q\mathcal{L}dt''}\right)Q\rho_{\rm tot}(0).
\end{aligned}
\tag{4}
$$

Here, $\rho_{\rm tot}(0)$ is the total density operator of the system and bath at initial time. The Liouville superoperators $\mathcal{L}_{\rm s}$, $\mathcal{L}_{\rm sb}$ and $\mathcal{L}_{\rm ren}$ are associated with corresponding Hamiltonian operators. Moreover, $\mathcal{L}_{\rm s}^{\rm eff}\cdot = -i[H_{\rm s} + H_{\rm ren}, \cdot]$ and $\mathcal{T}$ is the time ordering operator [10]. Next, we expand the correlated thermal equilibrium state up to the first order in the system-bath coupling and obtain

$$\rho^{\rm eq} \approx \frac{1}{Z_{\rm s}}\frac{1}{Z_{\rm b}}e^{-\beta(H_{\rm s}+H_{\rm b})} - \frac{1}{Z_{\rm s}}\frac{1}{Z_{\rm b}}\int_0^{\beta} d\beta' e^{-(\beta-\beta')(H_{\rm s}+H_{\rm b})}H_{\rm sb}^{(1)}e^{-\beta'(H_{\rm s}+H_{\rm b})}, \tag{5}$$

with the respective partition functions $Z_{\text{tot}} = \text{tr}\exp(-\beta H_{\text{tot}})$, $Z_{\text{b}} = \text{tr}_{\text{b}}\exp(-\beta H_{\text{b}})$, and $Z_{\text{s}} = \text{tr}_{\text{s}}\exp(-\beta H_{\text{s}})$. Next, we take the trace over the system degrees of freedom on both sides of Eq. (5) and get

$$\rho_{\text{b}}^{\text{eq}} = \frac{1}{Z_{\text{b}}} e^{-\beta H_{\text{b}}} + \frac{\chi}{Z_{\text{b}}} \int_0^\beta d\beta' e^{-(\beta-\beta')H_{\text{b}}} \left(\sum_{i=1}^N c_i x_i \right) e^{-\beta' H_{\text{b}}} . \tag{6}$$

Here, $\chi = (1/Z_{\text{s}})\text{tr}_{\text{s}} \left[f(x) e^{-\beta H_{\text{s}}} \right]$ with the coupling function $f(x)$ defined below Eq. (3).

As well established, the bath and coupling parameters can be collected in the spectral density

$$J(\omega) = \frac{\pi}{2} \sum_{j=1}^N \frac{c_j^2}{m_j \omega_j} \delta(\omega - \omega_j) . \tag{7}$$

This enters in the bath correlation function

$$c(t) = \int_{-\infty}^{\infty} \frac{d\omega}{2\pi} J(\omega) \cos(\omega t) \coth\left(\frac{\beta\omega}{2}\right) - i \int_{-\infty}^{\infty} \frac{d\omega}{2\pi} J(\omega) \sin(\omega t) \equiv a(t) - ib(t) \tag{8}$$

with the real part $a(t)$ and imaginary part $b(t)$. After inserting Eqs. (5) and (6) into Eq. (4), we express the last three terms of Eq. (4) by $a(t)$ and $b(t)$ in the form

$$\begin{aligned} \mathcal{L}_{\text{s}}^{\text{eff}} &= \mathcal{L}_{\text{s}} + \mu \mathcal{L}_{\text{ren,s}} + \chi \mu \mathcal{L}^- , \\ K(t,t') &= \mathcal{L}^- \left(a(t-t') \mathcal{T} e^{\int_{t'}^t \mathcal{L}_{\text{s}}} \mathcal{L}^- + b(t-t') \mathcal{T} e^{\int_{t'}^t \mathcal{L}_{\text{s}}} \mathcal{L}^+ \right) , \\ \Gamma(t) &= \mathcal{L}^- \int_{-\infty}^0 dt' \left[a(t-t') \mathcal{T} e^{\int_{t'}^t \mathcal{L}_{\text{s}}} \mathcal{L}^- \rho_{\text{s}}^{\text{eq}} + b(t-t') \mathcal{T} e^{\int_{t'}^t \mathcal{L}_{\text{s}}} \mathcal{L}^+ \rho_{\text{s}}^{\text{eq}} \right] , \end{aligned} \tag{9}$$

with $\mathcal{L}^- = -i[H_{\text{sb}}, \cdot]$ and $\mathcal{L}^+ = [H_{\text{sb}}, \cdot]_+ - 2\chi$. The potential renormalization is given in terms of the spectral density as $\mu = \int_{-\infty}^{\infty} \frac{d\omega}{2\pi} J(\omega)/\omega$.

In order to obtain an analytic form of the bath correlation function, any given spectral density (in our particular case, we use the standard Ohmic form) can be approximated by a sum of Lorentzian-like spectral terms [11, 12] according to

$$J(\omega) = \frac{\pi}{2} \sum_{k=1}^n \frac{p_k \omega}{[(\omega + \Omega_k)^2 + \Gamma_k^2][(\omega - \Omega_k)^2 + \Gamma_k^2]} . \tag{10}$$

The spectral amplitude p_k, the frequency Ω_k and the width Γ_k follow from the expansion of the original function in terms of the Lorentzian shapes. Inserting the expanded form of $J(\omega)$ into Eq. (8) results in

$$\begin{aligned} a(t) &= \sum_{k=1}^n \frac{p_k}{8\Omega_k \Gamma_k} \coth\left[\frac{\beta}{2} (\Omega_k + i\Gamma_k) e^{i\Omega_k t - \Gamma_k t} \right] + \sum_{k=1}^n \frac{p_k}{8\Omega_k \Gamma_k} \coth\left[\frac{\beta}{2} (\Omega_k - i\Gamma_k) e^{-i\Omega_k t - \Gamma_k t} \right] + \frac{2i}{\beta} \sum_{k=1}^{n'} J(i\nu_k) e^{-\nu_k t}, \\ b(t) &= \sum_{k=1}^n \frac{ip_k}{8\Omega_k \Gamma_k} \left(e^{i\Omega_k t - \Gamma_k t} - e^{-i\Omega_k t - \Gamma_k t} \right) , \end{aligned} \tag{11}$$

with the Matsubara frequencies $\nu_k = 2\pi k/\beta$. Moreover, n' is the number of Matsubara frequencies used.

Next, we rewrite the correlation functions as $a(t) = \sum_{k=1}^{n_r} \alpha_k^r e^{\gamma_k^r t}$ and $b(t) = \sum_{k=1}^{n_i} \alpha_k^i e^{\gamma_k^i t}$ with $n_i = 2n$, $n_r = 2n+n'$. Then, we define new auxiliary "density matrices" which incorporate both memory effects and initial correlations according to

$$\begin{aligned} \rho_k^r(t) &= \left(\mathcal{T} e^{\int_0^t dt' \mathcal{L}_{\text{s}}} e^{\gamma_k^r t} \int_0^\infty dt' e^{\mathcal{L}_{\text{s}} t'} e^{\gamma_k^r t'} \mathcal{L}^- \rho_{\text{s}}^{\text{eq}} + \int_0^t dt' e^{\gamma_k^r (t-t')} \mathcal{T} e^{\int_{t'}^t L_{\text{s}}} \mathcal{L}^- \rho_{\text{s}}(t') \right) , \\ \rho_k^i(t) &= \left(\mathcal{T} e^{\int_0^t dt' \mathcal{L}_{\text{s}}} e^{\gamma_k^i t} \int_0^\infty dt' e^{\mathcal{L}_{\text{s}} t'} e^{\gamma_k^i t'} \mathcal{L}^+ \rho_s^{\text{eq}} + \int_0^t e^{\gamma_k^i (t-t')} \mathcal{T} e^{\int_{t'}^t dt' L_{\text{s}}} \mathcal{L}^+ \rho_{\text{s}}(t') \right) . \end{aligned} \tag{12}$$

The time-retarded Eq. (4) (first term) can be then deconvoluted into a set of coupled first-order equations as

$$\begin{aligned} \dot{\rho}_{\text{s}}(t) &= \mathcal{L}_{\text{s}}^{\text{eff}}(t) \rho_{\text{s}}(t) + \left[\sum_{k=1}^{n_r} \alpha_k^r \mathcal{L}^- \rho_k^r(t) + \sum_{k=1}^{n_i} \alpha_k^i \mathcal{L}^- \rho_k^i(t) \right] , \\ \dot{\rho}_k^r(t) &= (\mathcal{L}_{\text{s}}(t) + \gamma_k^r) \rho_k^r(t) + \mathcal{L}^- \rho_{\text{s}}(t), \qquad k = 1, \ldots, n_r, \\ \dot{\rho}_k^i(t) &= (\mathcal{L}_{\text{s}}(t) + \gamma_k^i) \rho_k^i(t) + \mathcal{L}^+ \rho_{\text{s}}(t), \qquad k = 1, \ldots, n_i. \end{aligned} \tag{13}$$

This set of coupled time local quantum master equations was used for the calculations of the quantum dynamics and the resulting spectra. It generates an effective time-nonlocal quantum dynamics due to the coupled differential equations.

B. Linear absorption and circular dichroism spectra

We have used the first-order transition dipole moment correlation function to calculate the absorption and CD spectra defined by

$$\begin{aligned} I(\omega) &\propto \omega \int_{-\infty}^{+\infty} dt e^{i\omega t} \langle \boldsymbol{\mu}(t)\boldsymbol{\mu}(0)\rangle_g, \\ CD(\omega) &\propto \omega \int_{-\infty}^{+\infty} dt e^{i\omega t} \langle \boldsymbol{R}_{m,n} \cdot \boldsymbol{\mu}_m \times \boldsymbol{\mu}_n(t)\rangle_g. \end{aligned} \tag{14}$$

Here, $\boldsymbol{\mu}$ is the transition dipole moment and the subscript g refers to performing the trace over a thermally equilibrated bath distribution. $\boldsymbol{R}_{m,n}$ is the distance vector between the monomers m and n. The correlation functions can be calculated as $\langle \boldsymbol{\mu}(t)\boldsymbol{\mu}(0)\rangle_g = \mathrm{tr_s}\{\boldsymbol{\mu}\mathrm{tr_b}[e^{-iHt}\boldsymbol{\mu}\rho_g e^{iHt}]\}$, and $\langle \boldsymbol{r}_{m,n}\cdot\boldsymbol{\mu}_m\times\boldsymbol{\mu}_n(t)\rangle_g = \mathrm{tr_s}\{\boldsymbol{R}_{m,n}\cdot\boldsymbol{\mu}_m\times\mathrm{tr_b}[e^{-iHt}\boldsymbol{\mu}_n\rho_g e^{iHt}]\}$.

C. Numerical calculations of two-dimensional spectra by highly parallelized message-passing-interface

For the calculation of two-dimensional spectra, we have applied the equation of motion-phase matching approach (EOM-PMA) established in Ref. [13]. In the EOM-PMA, the induced polarization in the direction of the photon-echo signal is calculated by the simultaneous propagation of three auxiliary density matrices $\rho_{i=1,2,3}(t)$, each of which obeys a modified effective equation of motion according to

$$\begin{aligned} \dot{\rho_1}(t) &= -i[H_s - V_1(t,t_1) - V_2^\dagger(t,t_2) - V_3^\dagger(t,t_3), \rho_1(t)] - \Re(t)\rho_1(t), \\ \dot{\rho_2}(t) &= -i[H_s - V_1(t,t_1) - V_2^\dagger(t,t_2), \rho_2(t)] - \Re(t)\rho_2(t), \\ \dot{\rho_3}(t) &= -i[H_s - V_1(t,t_1) - V_3^\dagger(t,t_3), \rho_3(t)] - \Re(t)\rho_3(t), \end{aligned} \tag{15}$$

where $V_\alpha(t,t_\alpha) = XAe^{-(t-t_\alpha)^2/(2\Gamma^2)}e^{i\omega t}$, X is the transition dipole operator, Γ is the pulse duration, and $\Re$ is a relaxation superoperator. All three above master equations are here calculated by adopting the TNL method of Eq. (13) to the auxiliary density operators with the corresponding different time-dependent Hamiltonians. Then, the third-order induced polarization is obtained as

$$P_{PE}(t_1,t_2,t_3,t) = e^{i\mathbf{k_s}\cdot\mathbf{r}}\langle X(\rho_1(t) - \rho_2(t) - \rho_3(t))\rangle + c.c., \tag{16}$$

where the brackets $\langle \ldots \rangle$ indicate the evaluation of the trace.

The total 2D Fourier-transformed spectrum is then given by the double Fourier transform of the photon-echo polarization signal with respect to the delay time $\tau = t_2 - t_1$ and t according to

$$S_{PE}(\omega_\tau, T, \omega_t) \sim \int_{-\infty}^{+\infty} d\tau \int_{-\infty}^{+\infty} dt e^{-i\omega_\tau\tau}e^{i\omega_t t}P_{PE}(\tau,T,t). \tag{17}$$

Here, ω_τ is the "coherence" frequency, ω_t is the "detection frequency", and $T = t_3 - t_2$ is the "waiting" time.

For the concrete simulation of the 2D electronic spectra of the PSII reaction center, a message-passing interface (MPI) has been used to minimize the computation time. The coherence time window $[-300\text{ fs}, 300\text{ fs}]$ was separated into time slices of length $d\tau = 10$ fs. For further simplification, the inhomogeneous broadening by the static disorder was simulated with more than 100 realizations and each realization was calculated by one CPU. Thus, the total number of CPUs we need for one 2D electronic spectrum at a given waiting time is $61 \times 100 = 6100$ CPUs.

III. CORRELATED DISTRIBUTION GENERATED BY THE CHOLESKY DECOMPOSITION

The Cholesky decomposition is used [14] to generate cross-correlated static disorder and bath fluctuations for the PSII reaction center. To do so, we first define a correlation matrix A which determines the degree and the type of

correlations between two elements. In our current model of the PSII reaction center, the correlation coefficients are given by

$$\begin{bmatrix} 1 & 0 & 0 & 0 & 0 & 0 & 0 & 0 & 0.5 & 0 & 0.125 & 0.125 \\ 0 & 1 & 0 & 0 & 0 & 0 & 0 & 0 & 0.5 & 0 & 0 & 0 \\ 0 & 0 & 1 & 0 & 0 & 0 & 0 & 0 & 0 & 0.125 & 0.125 & 0 \\ 0 & 0 & 0 & 1 & 0 & 0 & 0 & 0 & 0 & 0 & 0 & 0 \\ 0 & 0 & 0 & 0 & 1 & 0 & 0 & 0 & 0 & 0.125 & 0 & 0.125 \\ 0 & 0 & 0 & 0 & 0 & 1 & 0 & 0 & 0 & 0 & 0 & 0 \\ 0 & 0 & 0 & 0 & 0 & 0 & 1 & 0 & 0 & 0 & 0 & 0 \\ 0 & 0 & 0 & 0 & 0 & 0 & 0 & 1 & 0 & 0 & 0 & 0 \\ 0.5 & 0.5 & 0 & 0 & 0 & 0 & 0 & 0 & 1 & 0 & -0.05 & -0.05 \\ 0 & 0 & 0.125 & 0 & 0.125 & 0 & 0 & 0 & 0 & 1 & -0.05 & 0.05 \\ 0.125 & 0 & 0.125 & 0 & 0 & 0 & 0 & 0 & -0.05 & -0.05 & 1 & 0.05 \\ 0.125 & 0 & 0 & 0 & 0.125 & 0 & 0 & 0 & -0.05 & 0.05 & 0.05 & 1 \end{bmatrix} . \tag{18}$$

From this, the L-matrix can be obtained by a Cholesky decomposition according to $A = LL^\dagger$. In order to generate spatially correlated static disorder, we define an array $(\Delta_1, \Delta_2, \ldots)$ with the dimension being given by the number of exciton sites. Each Δ_i indicates the disorder for the ith site and is independently distributed according to a Gaussian. Similarly, to generate correlated bath fluctuations, we just need to define an array of fully independent baths, $(\sum_i x_i, \sum_j x_j, \ldots)$. Then, the combined correlated static disorder and bath fluctuations are obtained as the product of the L-matrix and the two independent arrays. This approach has been successfully applied to generate correlated noise in a system-bath model [14].

IV. MULTIPLE PATHWAYS OF THE CHARGE DYNAMICS IN A TETRAMER

In this section, we report the results of the calculated 2D electronic spectra up to 12 ps for a tetramer which includes two CT states. Again, the TNL method is used. In order to describe the multiple pathways of the CT dynamics, we include two CT states with the site energies 0 cm^{-1} and -500 cm^{-1}. Then, the system Hamiltonian is given by

$$H_{\text{tetra}} = \begin{bmatrix} 270 & 150 & -42 & -6 & 0 & 0 \\ 150 & 210 & -56 & 20 & 0 & 0 \\ -42 & -56 & 20 & 46 & 50 & 0 \\ -6 & 20 & 46 & 155 & 50 & 0 \\ 0 & 0 & 50 & 50 & 0 & 30 \\ 0 & 0 & 0 & 0 & 30 & -500 \end{bmatrix} \text{cm}^{-1} . \tag{19}$$

As before, we have analyzed the calculated 2D spectra for different waiting times by the global fitting approach. Four different decay-associated spectra (DAS) can be resolved, with the associated decay time constants 39 fs, 422 fs, 3.04 ps and ∞, see Fig. 1. The fastest process with the time constant 39 fs is the fast electronic dephasing. The two other components with 422 fs and 3.04 ps reflect the evidence of the charge separation process which correspond to the CT from the excitonic states to the CS state with site energy 0 cm^{-1}, and, the secondary charge separation process from the site with 0 cm^{-1} to -500 cm^{-1}. This proves that the multiple pathways of the charge separation process in a tetramer can be fully revealed by the 2D electronic spectroscopy upon using the 2DDAS.

V. LONG-LIVED VIBRATIONAL COHERENCE IN THE PSII REACTION CENTER

Finally, we address the dynamics observed in our measurement of the PSII reaction center. In Fig. 2, we present the extracted time traces of the four selected peaks A, B, C and D, as marked in the 2D electronic spectrum at the waiting time $T = 0$fs. We clearly observe long-lived oscillatory signals which last over the full available window of the measurment time of 2 ps at the room temperature. This observation is in agreement with the previous observations reported in Refs. [15, 16]. Based on our previous study [17], these small-amplitude oscillations are induced by ordinary vibrational coherence.

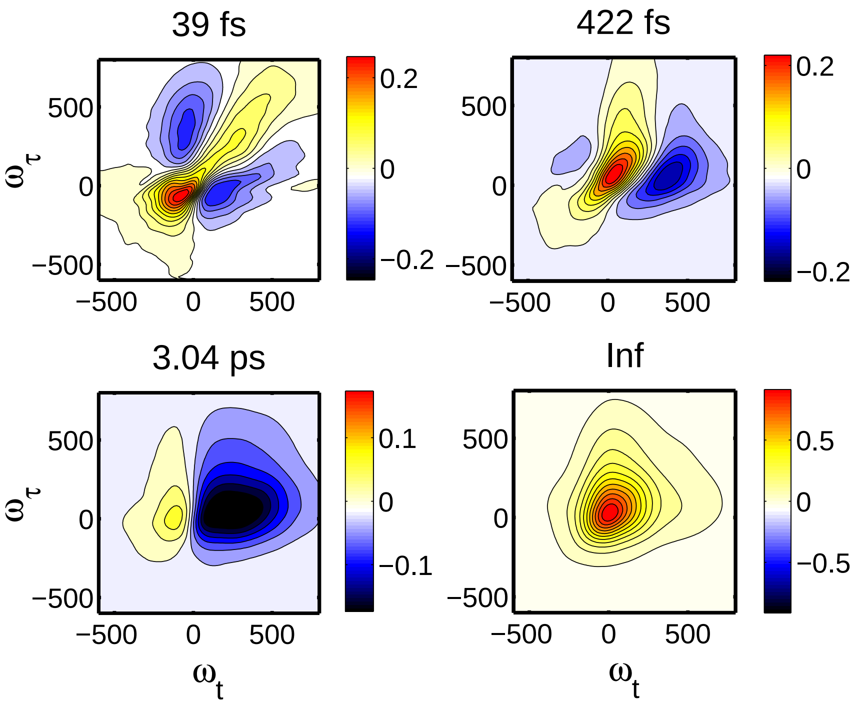

FIG. 1. The two-dimensional decay-associated spectra of a tetramer with two CS states included obtained from a global fitting approach. The two components with time constants 422 fs and 3.04 ps show the evidence of the charge transfer.

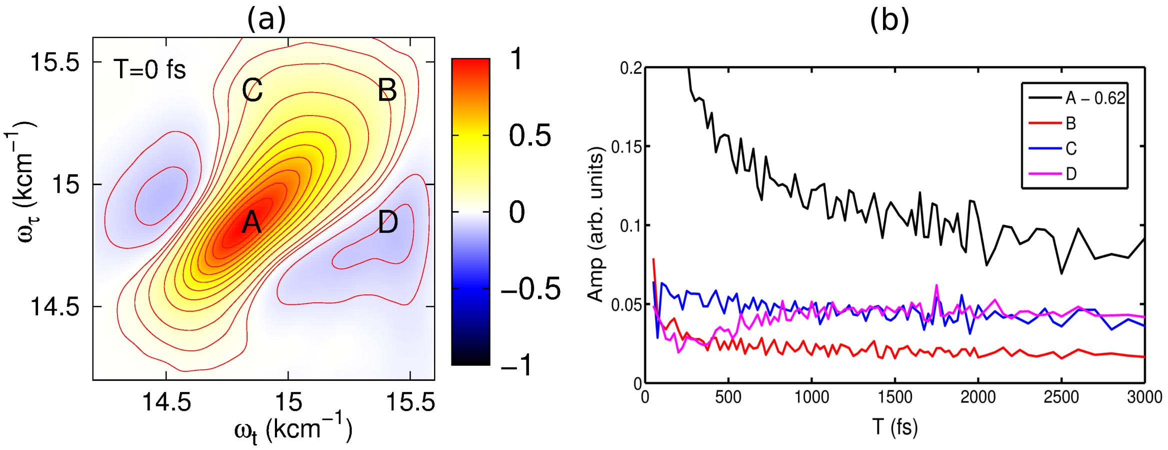

FIG. 2. (a) Experimentally measured 2D electronic spectrum of the PSII reaction center at waiting time $T = 0$ fs at room temperature. Four selected peaks are labeled by A, B, C and D in the 2D map. (b) Time evolution of the four selected peaks. In order to present the traces in one plot, the A-trace has been shifted downwards by -0.62. All the traces start at $T = 50$fs.

VI. DECAY-ASSOCIATED SPECTRUM OF 13.9 PS

We have experimentally resolved one decay component with a time scale of 13.9 ps, which can not be reproduced by the theoretical calculation, which is due to the required large, but unavailable computational resources. The resolved decay-associated spectrum is shown in Fig. 3, it only contains one diagonal peak at $\omega_\tau = \omega_t = 14800$ cm^{-1}.

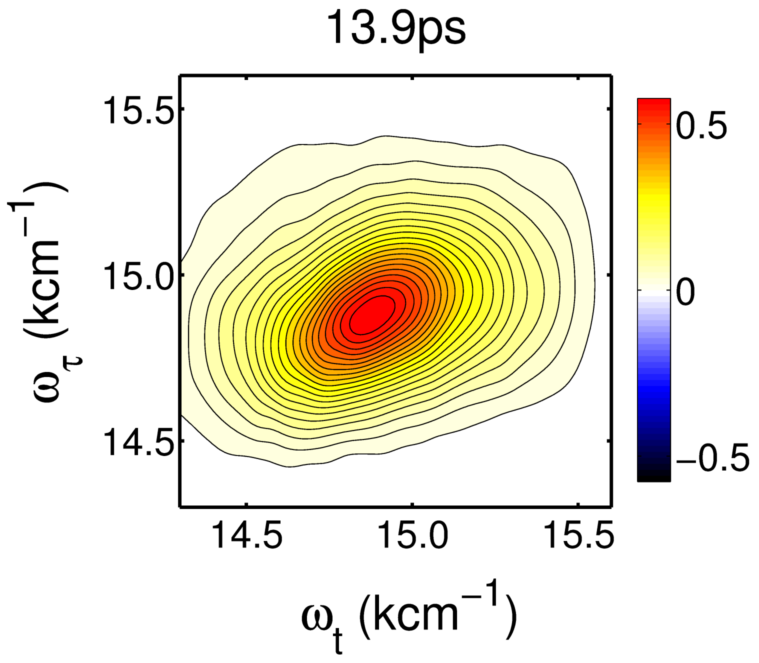

FIG. 3. The decay-associated spectrum (13.9 ps) resolved from the 2D spectroscopic measurement.

[1] Z. F. Liu, H. C. Yan, K. B. Wang, T. Y. Kuang, J. P. Zhang, L. L. Gui, X. M. An, and W. R. Chang, Nature **428**, 287 (2004).
[2] R. Standfuss, A. C. T. van Scheltinga, M. lamborghimi, and W. Kuhlbrandt, EMBO J. **24**, 919 (2005).
[3] Y. Umena, K. Kawakami, J. R. Shen, and N. Kamiya, Nature **473**, 55 (2011).
[4] A. Gelzinis, L. Valkunas, F. D. Fuller, J. P. Ogilvie, S. Mukamel, and D. Abramavicius, New J. Phys. **15**, 075013 (2013).
[5] E. Romero, I. H. M. van Stokkum, V. I. Novoderezhkin, J. P. Dekker, and R. van Grondelle, Biochem. **49**, 4300 (2010).
[6] V. I. Novoderezhkin, E. Romero, J. P. Dekker, and R. van Grondelle, Chem. Phys. Chem. **12**, 681 (2011).
[7] C. Meier, and D. J. Tannor, J. Chem. Phys. **111**, 3365 (1999).
[8] U. Kleinekathöfer, J. Chem. Phys. **121**, 2505 (2004).
[9] Zwanzig, R. *Lectures in Theoretical Physics*, Boulder, Colorado (Interscience, New York, 1961), Vol. 3.
[10] M. Morillo and R. I. Cukier, Phys. Rev. B **54**, 13962 (1997).
[11] U. Kleinekathöfer, I. Barvík, P. Heřman, I. Kondov, and M. J. Schreiber, Phys. Chem. B **107**, 14094 (2003).
[12] G. Ritschel and A. Eisfeld, J. Chem. Phys. **141**, 094101 (2014).
[13] M. F. Gelin, D. Egorova, and W. Domcke, J. Chem. Phys. **123**, 164112 (2005).
[14] A. G. Dijkstra, C. Wang, J. S. Cao, and G. R. Fleming, J. Phys. Chem. Lett. **6**, 627 (2015).
[15] F. D. Fuller, J. Pan, A. Gelzinis, V. Butkus, S. S. Senlik, D. E. Wilcox, C. F. Yocum, L. Valkunas, D. Abramavicius, and J. P. Ogilvie, Nature Chem. **6**, 706 (2014).
[16] E. Romero, R. Augulis, V. I. Novoderezhkin, M. Ferretti, J. Thieme, D. Zigmantas, and R. van Grondelle, Nature Phys. **10**, 676 (2014).
[17] H.-G. Duan, P. Nalbach, V. I. Prokhorenko, S. Mukamel, and M. Thorwart, New J. Phys. **17**, 072002 (2015).
